# Single-cell transcriptomics reveals cellular dynamics and chemokine CXCL2-mediated smooth muscle cell proliferation in arterial repair

**DOI:** 10.3389/fimmu.2025.1591557

**Published:** 2025-05-20

**Authors:** Xingxiao Huang, Yan Ping, Qiuli Sun, Mingjun Yu, Chao Yang, Xiao Liu, Long Wang, Jinyu Huang

**Affiliations:** ^1^ Hangzhou First People’s Hospital, Zhejiang University School of Medicine, Hangzhou, China; ^2^ Department of Cardiology, Westlake Laboratory of Life Sciences and Biomedicine, Affiliated Hangzhou First People’s Hospital, Westlake University School of Medicine, Hangzhou, China

**Keywords:** single-cell transcriptomics, arterial repair, smooth muscle cell proliferation, cellular dynamics, CXCL2

## Abstract

**Background:**

Percutaneous coronary intervention (PCI) remains the primary treatment for coronary artery disease (CAD), yet post-procedural arterial injury triggers cellular change and pathological inflammation, leading to thrombosis and restenosis. Recent studies have highlighted the chemokine CXCL2 play an important role in the immune response to tissue repair. However, the cellular mechanisms and the role of chemokine CXCL2 underlying arterial repair after PCI remain poorly understood.

**Methods:**

Single-cell RNA sequencing (scRNA-seq) was used to characterize the heterogeneity and gene expression profiles of cells in a femoral artery injury (FAI) model. Animal models of FAI and cellular experiments were used to validate the effects of CXCL2 on smooth muscle cell proliferation.

**Results:**

(1) Mesenchymal stem cells (MSCs), smooth muscle cells (SMCs), and macrophages play pivotal roles in arterial repair. And distinct subpopulations within these cell types were identified, each exhibiting unique functional characteristics and temporal dynamics during repair. (2) Notably, we identified an inflammatory SMC subpopulation (SMC2) that actively secretes chemokine CXCL2, which promotes SMC proliferation and mediates arterial remodeling after injury, suggesting its potential as a therapeutic target.

**Conclusions:**

This study provides a comprehensive cell atlas of the injured artery, offering valuable insights into the complex cellular interactions, signaling pathways and immune responses involved in orchestrating vascular repair. Our findings show chemokine CXCL2 as a key mediator of SMC proliferation and provide a roadmap for developing CXCL2-targeted therapies to improve vascular outcomes in PCI patients.

## 1 Introduction

Coronary artery disease (CAD) remains one of the foremost causes of morbidity and mortality worldwide, exerting a significant burden on healthcare systems and patient quality of life (1, 2). Percutaneous coronary intervention (PCI), which comprises balloon angioplasty and stent deployment, stands as a cornerstone therapy for CAD by mechanically dilating occluded vessels and restoring blood flow (3, 4). However, despite substantial technological advances in stent design—including drug-eluting stents—and adjunctive pharmacotherapy, adverse events such as thrombosis and in-stent restenosis continue to pose major clinical challenges (5, 6). These complications not only elevate the risk of further ischemic events but also necessitate additional interventions, underscoring the importance of understanding the underlying mechanisms that drive pathological vascular remodeling.

A critical gap in current knowledge pertains to the cellular and molecular processes that govern arterial injury and subsequent repair following PCI. Although prior investigations using bulk transcriptomic and proteomic analyses have offered valuable insights into global gene and protein expression shifts associated with vascular injury, such approaches are inherently limited in their capacity to dissect cell type–specific changes (7–9). The vascular wall is composed of multiple cell populations—including endothelial cells (ECs), smooth muscle cells (SMCs), and various immune cells—that respond differently to injury stimuli (10–13). Moreover, these cell types can transition into diverse phenotypic states under pathological conditions, complicating efforts to pinpoint precise therapeutic targets (11, 14). Consequently, a detailed understanding of the cellular heterogeneity and dynamic remodeling events at single-cell resolution is paramount for developing more effective interventions.

ScRNA-seq has revolutionized the field of vascular biology by enabling high-resolution profiling of distinct cell populations, their phenotypic states, and intercellular communication networks (15–17). Through scRNA-seq, researchers can capture the temporal evolution of injury responses—ranging from immediate inflammatory events to longer-term reparative and remodeling phases—and identify critical transcriptional regulators that orchestrate these processes (18, 19). In addition, scRNA-seq holds promise for unveiling previously unrecognized or rare cell subpopulations, including specialized immune subsets or resident stem/progenitor cells, which may be pivotal in mediating vascular healing (20, 21).

Emerging evidence implicates chemokine-mediated inflammatory signaling as a critical modulator of vascular repair processes (22). Among these mediators, CXCL2 has garnered particular interest due to its dual role in coordinating immune responses and regulating tissue remodeling (23, 24). As a potent chemoattractant, CXCL2 drives neutrophil infiltration through CXCR2 receptor activation, initiating acute inflammatory cascades that paradoxically contribute to both tissue damage and repair initiation (25). At present, the role of CXCL2 in vascular repair has not been reported.

In this study, we utilized scRNA-seq datasets obtained from a FAI model to delineate the dynamic cellular and molecular landscape following vascular damage. By constructing a comprehensive “cell atlas” of both normal and injured vessels, we aimed to uncover how major vascular cell populations—such as ECs, SMCs, and mesenchymal stem cells (MSCs)—undergo phenotypic and functional reprogramming in response to injury. Notably, our analyses revealed the emergence of specialized immune subpopulations, including distinct macrophage subsets, that appear to orchestrate critical aspects of tissue repair. Furthermore, we discovered that CXCL2 secreted by SMCs not only promotes their own proliferation but also potentially influences the broader repair milieu. Importantly, SMC proliferation was attenuated upon inhibition of CXCL2, suggesting a direct link between CXCL2 signaling and pathological vascular remodeling. Collectively, these findings provide mechanistic insights into the temporal evolution of arterial repair processes, offering new avenues for therapeutic intervention aimed at optimizing PCI outcomes. By delineating the heterogeneity and complexity of the vascular response to injury at single-cell resolution, this work highlights potential targets for pharmacological modulation that could mitigate thrombosis, reduce restenosis, and ultimately improve long-term vascular health.

## 2 Materials and methods

### 2.1 Data source

This study is a secondary analysis of publicly available single-cell RNA sequencing (scRNA-seq) data from the Xu et al.'s study, which investigated femoral artery injury (FAI) in an animal model. We obtained the raw scRNA-seq data from the Gene Expression Omnibus (GSE182232). All analyses described below were performed in accordance with the repository’s data usage terms and any relevant license or data-sharing agreements.

### 2.2 Data preprocessing and quality control

The raw count matrices were imported into R (4.2.1) for further processing. Quality control steps were conducted using the Seurat package (version 4.1) to remove low-quality cells and potential doublets. Cells with fewer than a minimum number of detected genes (200) or an excessively high number of unique molecular identifiers (UMIs) likely representing doublets were excluded. Cells with a high percentage of mitochondrial gene expression (>15%) were also removed to avoid capturing dying or stressed cells. Genes that were not detected in a sufficient number of cells across the dataset (fewer than 3 cells) were excluded to reduce noise.

### 2.3 Data normalization

After filtering, gene expression values were normalized using a log normalization method implemented in Seurat. The normalized data were then scaled to unit variance and zero-mean for downstream analyses. In cases where more robust correction was required, we employed Harmony to align cells across three time points.

### 2.4 Dimensionality reduction and unsupervised clustering

A principal component analysis was performed on the scaled data to reduce dimensionality. Significant principal components (PCs) were selected based on the Elbow Plot or Jack Straw methods in Seurat. The first several significant PCs (30 PCs) were used for non-linear dimensionality reduction via UMAP (Uniform Manifold Approximation and Projection). Cells were clustered using a graph-based approach (Seurat’s “FindClusters” function) to identify discrete cell populations. The resolution parameter was tuned to achieve biologically meaningful clustering.

### 2.5 Cell type annotation and subpopulation analysis

Differential expression analysis (Wilcoxon rank-sum test) was performed to identify cluster-specific marker genes. Known marker genes from the literature (*Acta2*, *Myh11* for smooth muscle cells; *Pecam1*, *Vwf* for endothelial cells; *Cd14*, *Adgre1* for macrophages) were used to annotate each cluster. For specific cell types of interest (macrophages, mesenchymal stem cells, smooth muscle cells), we isolated and re-clustered these populations to resolve finer subpopulations (Mac1, Mac2, Mac3; MSC1, MSC2, MSC3; SMC1, SMC2, SMC3, SMC4).

### 2.6 Functional and pathway enrichment

Differential Expression Across Conditions: We compared gene expression patterns among Control, FAI 2w, and FAI 4w groups using the “FindMarkers” function in Seurat. Significantly up- or downregulated genes were subjected to pathway enrichment analyses using Kyoto Encyclopedia of Genes and Genomes (KEGG), Gene Ontology (GO), or Gene Set Enrichment Analysis (GSEA). This revealed key biological processes (oxidative phosphorylation, cardiac muscle contraction, MAPK signaling) relevant to vascular injury and repair. CellChat or a similar pipeline was employed to analyze ligand–receptor interactions among different cell types or subtypes. Specific signaling pathways (PDGF, TNF, TGFβ, SPP1, CHEMERIN) were examined in detail. Chord diagrams and bubble plots were used to illustrate changes in intercellular signaling across time points.

### 2.7 Pseudotime trajectory analysis

To explore the potential lineage progression within macrophage and smooth muscle cell subsets, we performed pseudotime trajectory inference using Monocle or Slingshot. Cells were ordered along a computational trajectory based on their transcriptomic profiles, enabling us to visualize potential transitions (Mac1 → Mac2/Mac3 SMC1 → SMC2/SMC3).

### 2.8 Cell experiment

Upon reaching 80% confluence, human umbilical vein endothelial cells (HUVECs) were subjected to mechanical disruption through cell curettage for half of the population, followed by three phosphate-buffered saline (PBS) washes and subsequent culture in standard medium (10% FBS, 1% Penicillin and Streptomycin in Dulbecco’s Modified Eagle Medium (DMEM)). The control group underwent medium replacement after PBS washing. Following a 24-hour incubation period, the cell supernatant was harvested and combined with DMEM in a 1:1 ratio. The upper clearance of intervention smooth muscle cells (SMCs) was categorized into four groups: Control HUVECs conditioned medium (CM), Injury HUVECs CM, Control HUVECs CM + Amodiaquine, and Injury HUVECs CM + Amodiaquine. Amodiaquine functioned as an inhibitor of CXCL2. Subsequent to a 24-hour intervention at a concentration of 10 μM, Cell Counting Kit-8 (CCK8) reagent was introduced, and absorbance was quantified at 450 nm utilizing a spectrophotometer after a 4-hour interval.

### 2.9 Femoral artery injury model

The femoral artery injury (FAI) model using wire insertion was implemented as previously outlined (26). In summary, 8-week-old male mice were anesthetized via intraperitoneal injection of 1.25% avertin(#75-80-9, Sigma) at 200 ul/10g dosage. The femoral artery was exposed by meticulously separating it from the adjacent vein and connective tissue using microsurgical instruments. A flexible 21-gauge wire measuring 0.25 mm in diameter (obtained from CROSS-IT 200XT guide wire tips, Abbott Laboratories, Illinois, USA) was introduced into the femoral artery and pushed 5–10 mm in the direction of the iliac artery, where it remained for 3 minutes. Following the ligation of the profunda femoris branch, blood flow was reestablished. After the procedure, the mice were placed on a heating pad until they fully regained consciousness and were euthanized at specified intervals for subsequent analysis.

### 2.10 Immunofluorescence

For histological examination, samples from the femoral artery were preserved in 10% formalin for 48 hours before being embedded in paraffin. The embedded samples were then cut into 5 μm thick sections. For immunofluorescence staining, the rehydrated sections were subjected to antigen retrieval using an antigen retrieval sodium citrate buffer (#C1013, Solarbio). The sections were subsequently blocked with PBST (consisting of 3% BSA (#4240GR100, Biofroxx) and 0.1% Triton X-100 (#9002-93-1, Solarbio) in PBS) at room temperature for 60 min. The sections were then incubated with primary antibodies overnight at 4°C. After washing the sections three times with PBS, they were incubated with Alexa Fluor-conjugated secondary antibodies for 60 min at room temperature. The sections were then mounted using a DAPI-containing antifade mounting medium (#G1407, Servicebio). The primary antibodies used were anti-α-SMA (#ab7817, Abcam, 1:500), CXCL2(#26791-1-AP, Proteintech, 1:500). Images were acquired using a Zeiss LSM 900 confocal microscope (Oberkochen, Germany).

### 2.11 RNA extraction and RT-qPCR

Total RNA was isolated from the indicated cells or tissues using TRIzol reagent (#15596018CN, Thermo Fisher). cDNA was generated using the PrimeScript™ RT Reagent Kit with gDNA Eraser (Perfect Real Time) (#RR047A, Takara). Real-time quantitative PCR was performed using SYBR Green PCR reagents (#AQ601, TransGen) and ABI QuantStudio 5. *Rps18* were used as internal controls for normalization.

### 2.12 Statistical analysis

Statistical analysis was carried out as indicated in the figure legends. All data are presented as the mean ± s.e.m. The differences were analyzed one-way analysis of variance (ANOVA). A *p* value less than 0.05 was considered statistically significant. All statistical analyses were performed using GraphPad Prism 8.0 (GraphPad Software).

## 3 Result

### 3.1 Global cell types and their changes in the femoral artery injury model revealed by scRNA-seq

**Figure 1:**
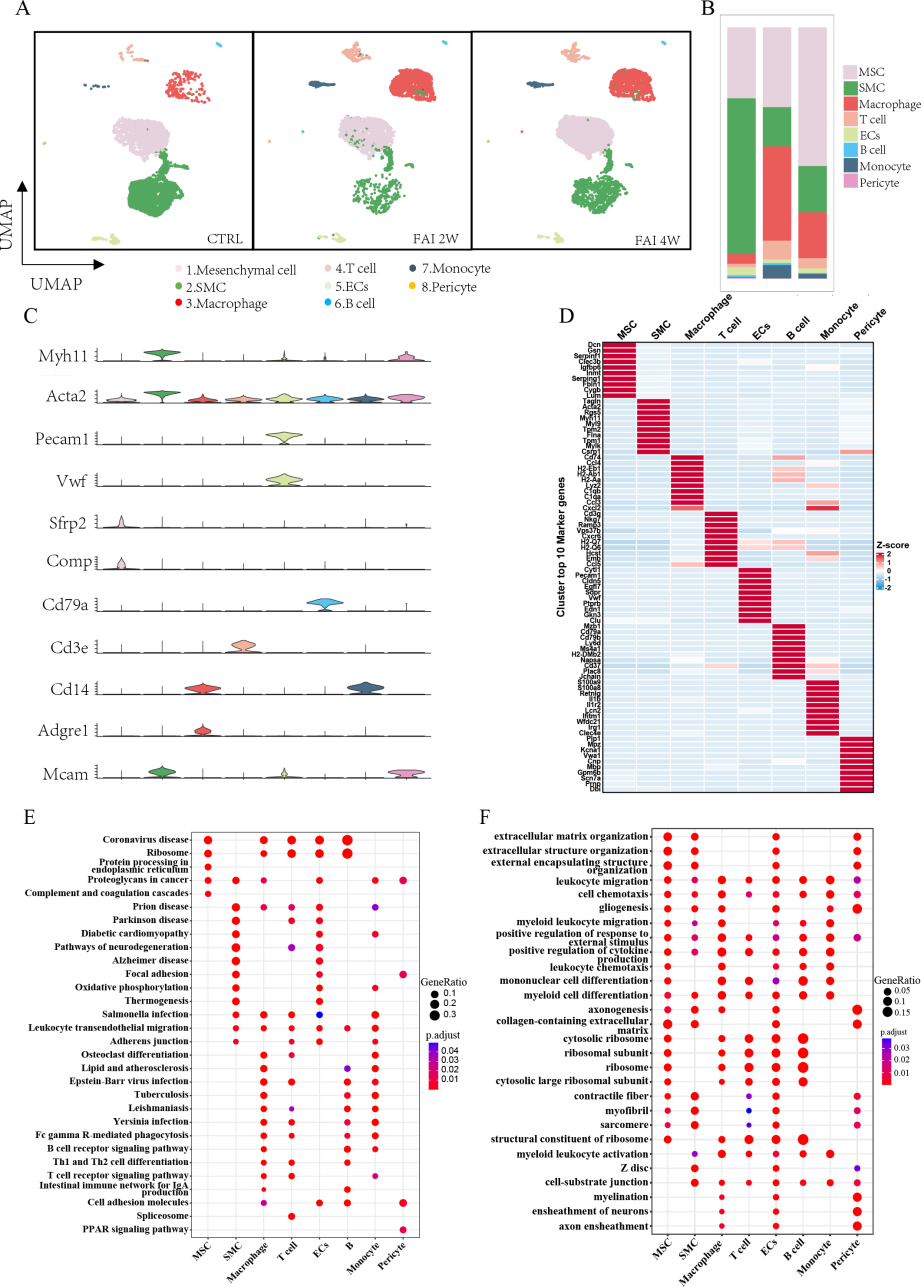
The global cell types and changes in femoral artery injury by scRNA-seq. **(A)** UMAP visualization of isolated femoral artery, based on 15996 single-cell transcriptomes pooled from control (CTRL-5192), femoral artery injury 2 weeks (FAI 2W-4115), and femoral artery injury 4 weeks (FAI 4W-6689). **(B)** Bar charts showing the proportion of major cell types and each cell clusters. **(C)** Violin plot of gene markers for differentiated cell in all clusters. **(D)** Heat maps show hypervariable genes in different cells. **(E, F)** GSEA enrichment analysis of different cell populations E (KEGG) F(GO).

We analyzed scRNA-seq data from the FAI model described in the Xu et al.'s study (26). In his study, arterial samples were collected at various time points, including uninjured arteries, as well as 2 and 4 weeks post-injury, for single-cell RNA sequencing (scRNA-seq). After quality control, unsupervised clustering identified eight distinct cell populations. Based on the expression of cell type–specific marker genes and top enriched genes, these clusters were classified as mesenchymal cells (MSCs; *Acta2*, *Sfrp2*, *Comp*), smooth muscle cells (SMCs; *Myh11*, *Acta2*), macrophages (*Cd14*, *Adgre1*), T cells (*Cd3e*), endothelial cells (ECs; *Pecam1*, *Vwf*), B cells (*Cd79a*), monocytes (*Cd14*), and pericytes (*Mcams*) (**Figures 1A, C**). To quantify changes in cell abundance, we compared the cell populations at different time points following FAI. After two weeks, we observed an increase in MSCs and immune cells—specifically macrophages, T cells, and monocytes—alongside a decrease in SMCs. In contrast, at four weeks, there was a marked rise in MSCs and SMCs and a reduction in immune cells compared to the two-week time point (**Figure 1B**). Each cluster exhibited distinct gene expression profiles (**Figure 1D**). Gene set enrichment analysis (GSEA) of hypervariable genes revealed cell type–specific functional pathways among these populations (**Figures 1E, F**). Our analyses revealed that immune cells following arterial injury exhibit pronounced engagement in cell adhesion and transmigration mechanisms, as evidenced by KEGG pathway enrichment for cell adhesion molecules, leukocyte transendothelial migration, and immune response activation. Complementary GO analysis further delineated their functional polarization toward extracellular matrix reorganization, cytokine secretory activity and cytoskeletal reprogramming, collectively underscoring their dual roles in inflammatory recruitment and tissue-remodeling cascades during vascular repair.

### 3.2 Complex intercellular communication network in the global cell population of femoral artery injury

**Figure 2:**
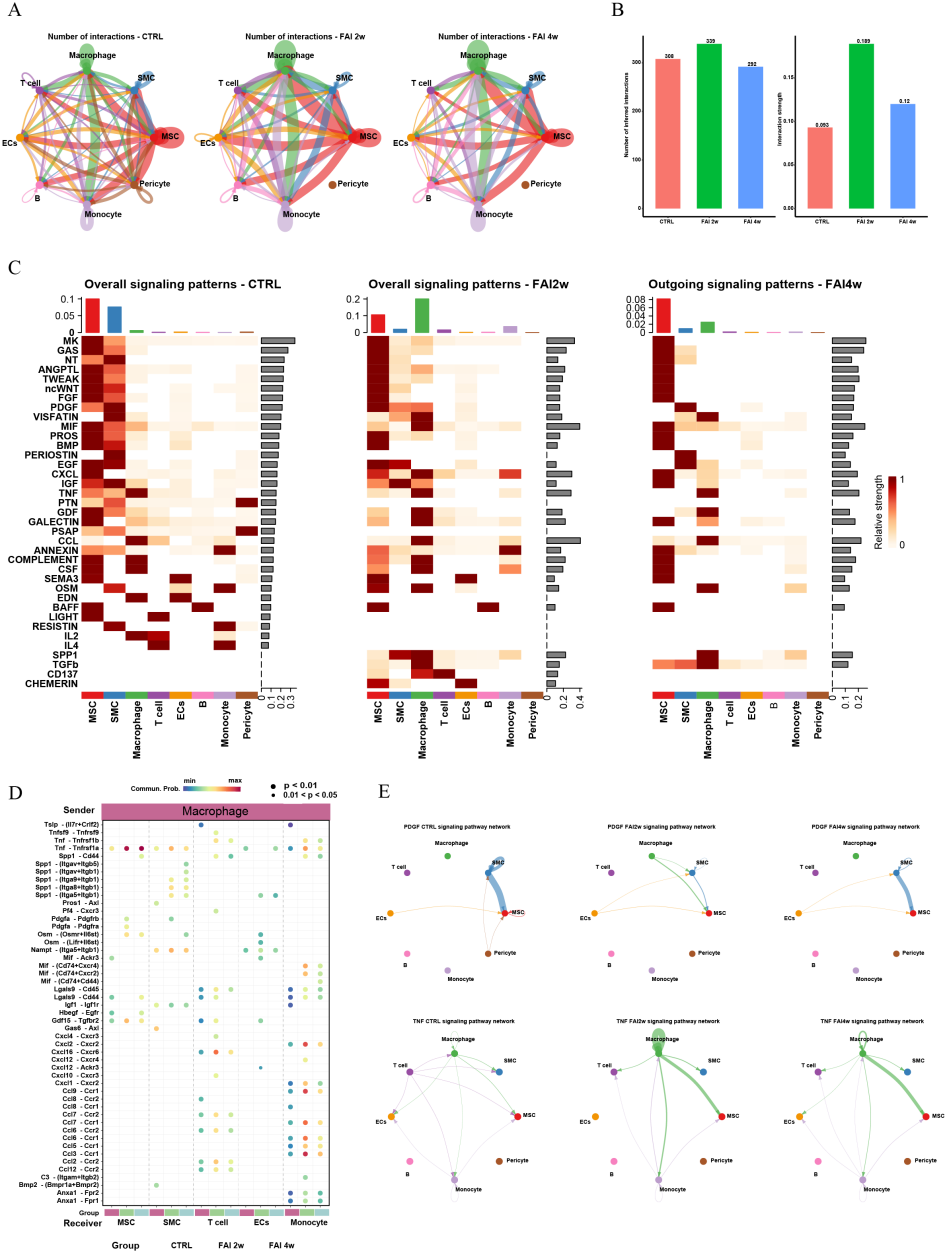
Complex intercellular communication network in the global cells in femoral artery injury mouse. **(A)** Network visualizing potential specific interactions in femoral artery control, FAI 2W and FAI 4W group, in which nodes are clusters and edges represent the number of significant ligand–receptor pairs. **(B)** Bar graphs showing changes in the number and strength of interactions of cellular communication between different cells in the normal and femoral artery injury groups. **(C)** Incoming and outcoming signaling pattern in in femoral artery control, FAI 2W and FAI 4W group. **(D)** Comparison of the significant ligand‐receptor pairs across different subtypes. Dot color represents the communication probability of the specific ligand‐receptor pair between sender cluster and receiver cluster. **(E)** Chordal plots showing differences in PDGF and TNF signaling pathway interactions between different cell populations in the control, FAI2w and FAI4W groups.

ScRNA-seq analysis also uncovered extensive interactions among MSCs, SMCs, ECs, and immune cells, which became more pronounced in response to endovascular injury. Notably, MSCs exhibited the most robust interactions with immune cells—particularly macrophages—and these interactions strengthened as tissue repair progressed (**Figures 2A, B**). As the repair process advanced, individual cell clusters displayed distinct patterns of incoming and outgoing signaling. A comprehensive analysis indicated that post-injury subpopulations collectively showed enhanced secretion of factors such as SPP1, TGFβ, CD137, and CHEMERIN, each playing a critical role at different stages of arterial injury (**Figure 2C**). Furthermore, an in-depth examination of prominent ligand–receptor pairs across various subtypes revealed temporal changes in intercellular signaling throughout the different phases of femoral artery injury repair (**Figure 2D**). Chord diagrams illustrated dynamic shifts in PDGF and TNF signaling pathways among distinct cell populations in control, FAI 2w, and FAI 4w groups (**Figure 2E**), underscoring the evolving nature of these intercellular interactions as arterial repair progressed.

### 3.3 Heterogeneity of mesenchymal cells in femoral artery injury

**Figure 3:**
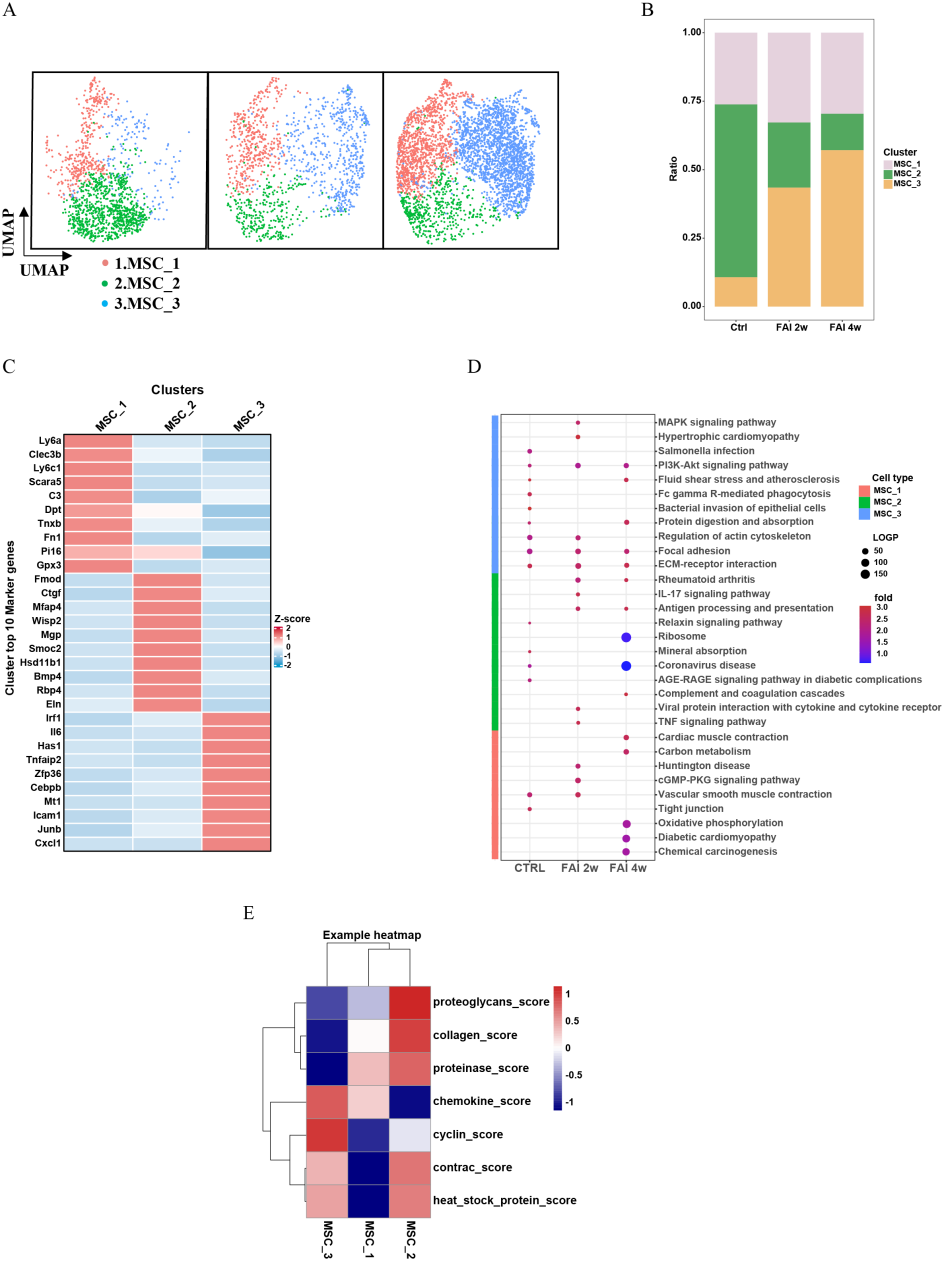
Heterogeneity of mesenchymal cells in femoral artery injury. **(A)** UMAP visualization of MSC isolated femoral artery control, femoral artery injury 2 weeks (FAI 2W), and femoral artery injury 4 weeks (FAI 4W). **(B)** Bar charts showing the proportion of MSC cell types and each cell clusters. **(C)** Heat maps show hypervariable genes in different cells. **(D)** The MSC subtypes KEGG dynamic changes in ctrl, FAI 2W and FAI 4W group. **(E)** Module scores of 7 features (or functions) in MSC subtypes.

To investigate the heterogeneity and functional roles of mesenchymal stem cells (MSCs) during vascular injury, we subdivided MSCs into three clusters—MSC1, MSC2, and MSC3—based on their top 10 differentially expressed marker genes (**Figures 3A, C**). At two weeks post-injury (FAI 2w), the abundance of MSC1 cells showed a slight increase, whereas MSC3 underwent a more pronounced expansion. By contrast, MSC2 exhibited a substantial decline compared to the control group (**Figure 3B**). At four weeks post-injury (FAI 4w), MSC1 experienced a modest decrease, MSC2 continued to diminish, and MSC3 maintained its upward trend (**Figure 3B**). Kyoto Encyclopedia of Genes and Genomes (KEGG) pathway analysis illustrated dynamic shifts in signaling pathways across these three clusters following FAI (**Figure 3D**). MSCs exhibit dynamic functional changes during femoral artery injury (FAI) repair, as evidenced by distinct pathway enrichment patterns at 2 weeks (FAI 2w) and 4 weeks (FAI 4w). MSC1 is enriched in the cGMP-PKG signaling and vascular smooth muscle contraction pathways at FAI 2w but not at FAI 4w, suggesting its role in sensing and adapting to early injury signals. Similarly, MSC3 shows heightened activity in pathways like MAPK/PI3K signaling, actin cytoskeleton regulation, and ECM-receptor interaction at FAI 2w, with reduced expression by FAI 4w, further supporting early-phase functional dominance. In contrast, MSC2 is enriched in immune-related pathways such as IL-17 signaling and antigen processing/presentation at FAI 2w, but these pathways decline by FAI 4w, aligning with diminished immune activity during later repair stages. Collectively, these findings highlight time-dependent MSC heterogeneity, where subsets like MSC1 and MSC3 drive adaptive responses early in repair, while MSC2 contributes to transient immune modulation that resolves by FAI 4w. Differential module score analyses further revealed distinct functional profiles among the MSC subpopulations (**Figure 3E**). In particular, MSC3 displayed higher chemokine expression and cyclin-related scores, indicative of a heightened capacity for both immune cell recruitment and proliferative activity. Notably, the absolute number and proportion of MSC3 cells increased significantly after arterial injury, suggesting a pivotal role in orchestrating immune responses and cellular proliferation during the repair process. Although MSC2 declined markedly post-injury, it retained strong collagen- and proteinase-related functions, underscoring its essential contribution to tissue remodeling and repair.

### 3.4 Dynamic changes in macrophages during femoral artery injury

**Figure 4:**
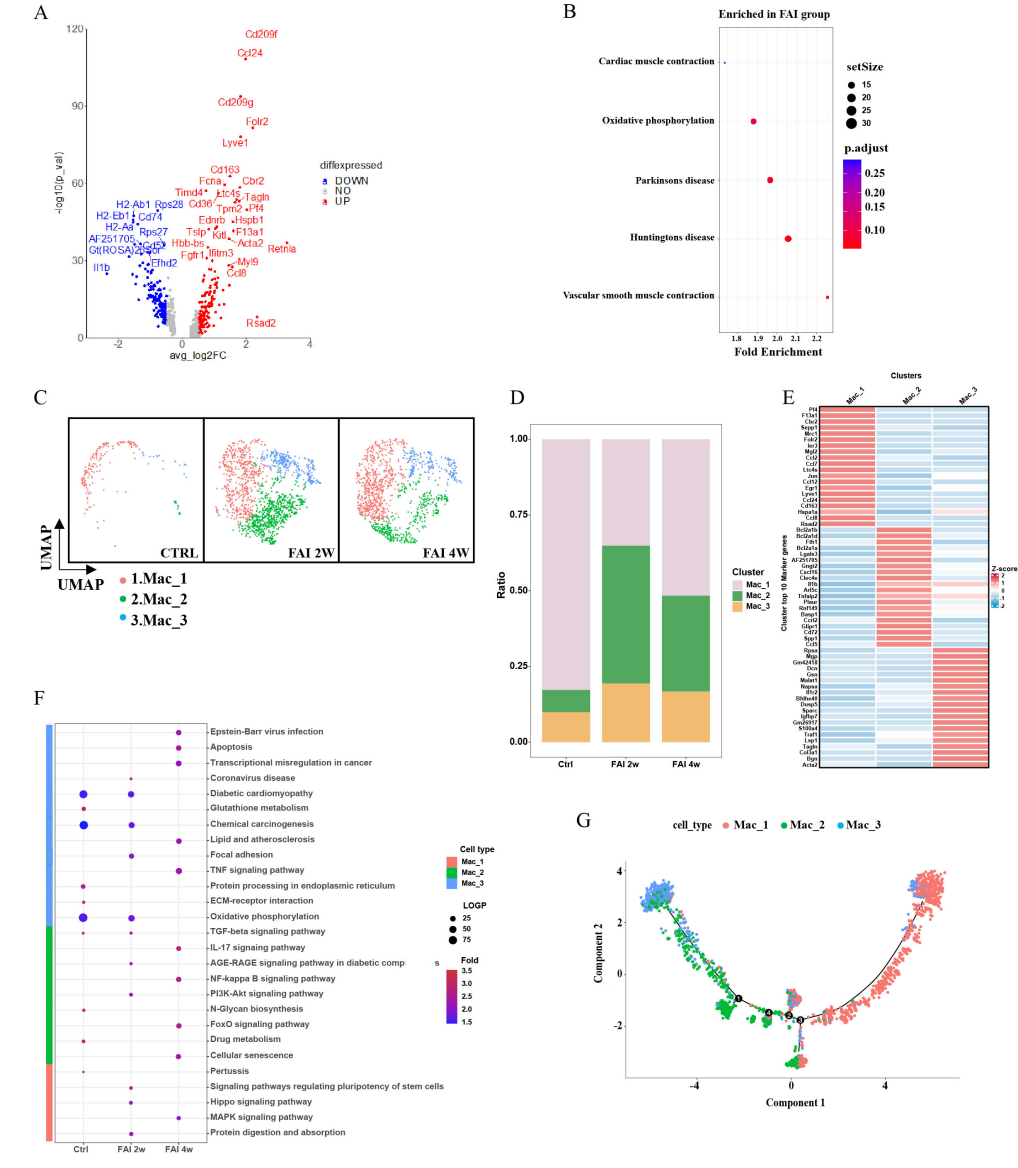
Dynamic change of macrophage in femoral artery injury. **(A)** Volcano plot showing differences in macrophages cell gene expression between control and femoral artery injury groups. **(B)** KEGG analysis for upregulated DEGs of total macrophages after femoral artery injury. **(C)** UMAP visualization of macrophages isolated femoral artery control, femoral artery injury 2 weeks (FAI 2W), and femoral artery injury 4 weeks (FAI 4W). **(D)** Bar charts showing the proportion of macrophages types and each cell clusters. **(E)** Heat maps show hypervariable genes in different cells. **(F)** The macrophage subtypes KEGG dynamic changes in ctrl, FAI 2W and FAI 4W group. **(G)** Pseudotime trajectory analysis of macrophage subtypes. Arrows represent the direction of the differentiation trajectory.

Our data indicate that macrophages are key mediators of vascular repair. Comparative gene expression analyses revealed distinct transcriptional signatures in macrophages from injured samples relative to controls (**Figure 4A**). KEGG pathway enrichment demonstrated that, following femoral artery injury (FAI), macrophages exhibited notable changes in pathways related to cardiac muscle contraction, oxidative phosphorylation and vascular smooth muscle contraction (**Figure 4B**). Subsequent clustering based on differentially expressed genes stratified macrophages into three groups: Mac1, Mac2, and Mac3 (**Figure 4E**). All three populations expanded in response to vascular injury (**Figure 4C**). However, the relative proportion of each subtype varied over time: at FAI 2w, Mac1 decreased, while Mac2 and Mac3 increased. By FAI 4w, Mac1 rebounded, whereas Mac2 and Mac3 declined compared to the two-week time point (**Figure 4D**). Further pathway enrichment analyses indicated that each macrophage subtype engaged in distinct functional pathways at different stages of injury repair (**Figure 4F**). We specifically focused on the Mac-2 subgroup which demonstrated the most significant changes in both proportion and absolute numbers following injury. Our pathway analysis revealed marked enrichment of Mac-2 subgroup in the NF-κB pathway, PI3K-AKT signaling, and FoxO signaling pathways. These molecular signatures indicate that Mac-2 macrophages undergo adaptive reprogramming under stress conditions, establishing a delicate balance between pro-inflammatory responses and apoptosis resistance to maintain cellular homeostasis during tissue repair. Notably, pseudotime trajectory analysis revealed a progression from Mac1 toward Mac2 and Mac3, providing insights into macrophage lineage dynamics and their respective contributions to the reparative process (**Figure 4G**).

### 3.5 Heterogeneity of smooth muscle cells in femoral artery injury

**Figure 5:**
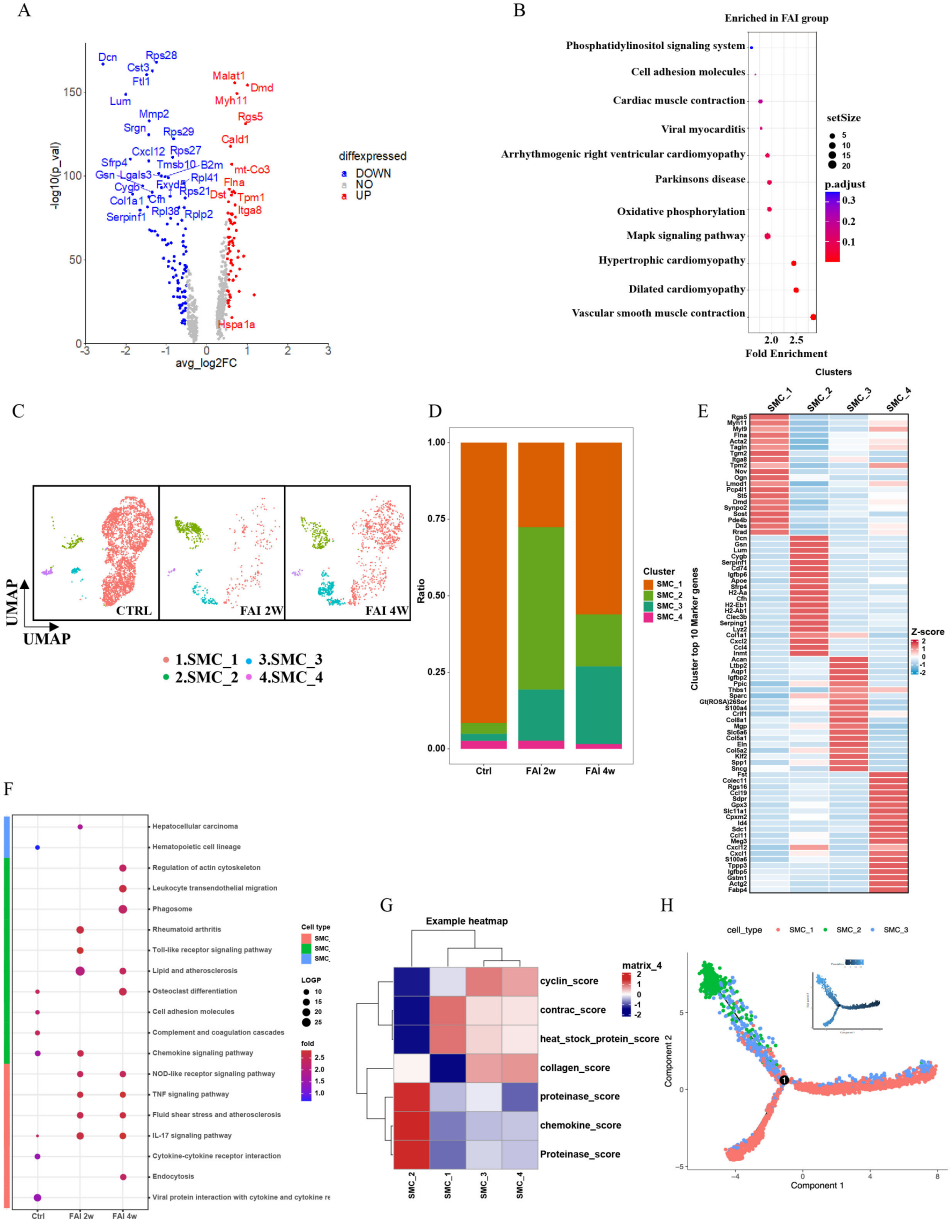
Heterogeneity of smooth muscle cells in femoral artery injury. **(A)** Volcano plot showing differences in SMC gene expression between control and femoral artery injury groups. **(B)** KEGG analysis for upregulated DEGs of total SMC after femoral artery injury. **(C)** UMAP visualization of SMC isolated femoral artery control, femoral artery injury 2 weeks (FAI 2W), and femoral artery injury 4 weeks (FAI 4W). **(D)** Bar charts showing the proportion of SMC cell types and each cell clusters. **(E)** Heat maps show hypervariable genes in different cells. **(F)** The SMC subtypes KEGG dynamic changes in ctrl, FAI 2W and FAI 4W group. **(G)** Module scores of 7 features (or functions) in SMC subtypes. **(H)** Pseudotime trajectory analysis of SMC subtypes. Arrows represent the direction of the differentiation trajectory.

To elucidate the role of smooth muscle cells (SMCs) in arterial repair, we first examined their gene expression profiles. Volcano plots showed that genes related to smooth muscle formation were upregulated in the FAI group (**Figure 5A**). Pathway analyses revealed that phosphatidylinositol signaling, cell adhesion molecule (CAM) pathways, cardiac muscle contraction, viral myocarditis, arrhythmogenic right ventricular cardiomyopathy, oxidative phosphorylation, MAPK signaling, hypertrophic cardiomyopathy, cardiomyopathy, and vascular smooth muscle contraction pathways were particularly enriched after injury (**Figure 5B**). We further classified SMCs into four subgroups—SMC1, SMC2, SMC3, and SMC4—based on distinct gene expression signatures (**Figure 5E**). At 2 weeks post-injury, the population of SMC1 decreased significantly, SMC4 showed a moderate reduction, and SMC2 and SMC3 both expanded (**Figure 5C**). Between 2 and 4 weeks, SMC1 and SMC3 continued to increase, while SMC2 declined, and SMC4 remained relatively stable (**Figure 5C**). Changes in both the proportion and total population mirrored these observations (**Figure 5D**). Pathway enrichment patterns also varied over time (**Figure 5F**). Among smooth muscle cell (SMC) subsets, SMC 2 displayed the most notable changes after femoral artery injury (FAI). At FAI 2w, SMC 2 was enriched in pathways such as chemokine signaling and Toll-like receptor signaling, identifying it as an immune-linked subset. By FAI 4w, these pathways showed reduced activity, reflecting diminished immune responses during later stages of repair. Feature module scores indicated that SMC2 exhibited distinct functional characteristics, including higher expression of proteinases and chemokines, suggesting a unique regulatory role in endovascular injury repair (**Figure 5G**). Notably, the proportion of SMC2 underwent the most pronounced shift following arterial injury, hinting at its involvement in modulating inflammation or promoting tissue regeneration. Pseudotime trajectory analysis supported this hypothesis, showing a transition from SMC1 toward SMC2 and SMC3 (**Figure 5H**).

### 3.6 CXCL2 mediates proliferation of smooth muscle cells

**Figure 6:**
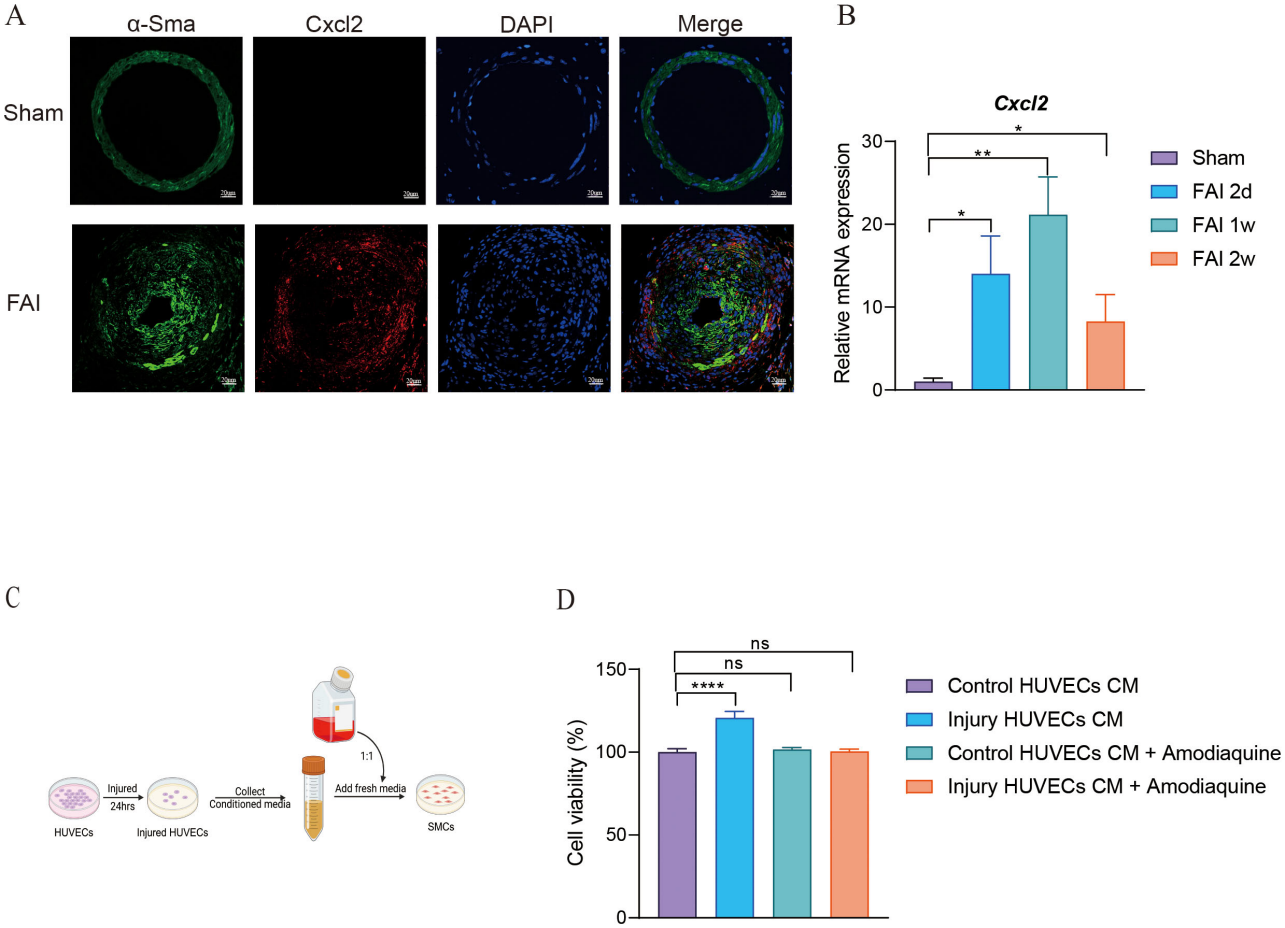
CXCL2 mediates proliferation of smooth muscle cells. **(A)** Immunofluorescence staining for α-SMA and CXCL2 in femoral artery sections after FAI 2w. Scale bar, 20um. **(B)** mRNA levels of CXCL2 in femoral artery sections at the indicated time points post-FAI. *Rps18* is used as an internal reference. **(C)** A schematic illustrating the experimental design. **(D)** Cell viability of SMCs. Data are all shown as the mean ± s.e.m. *p < 0.05 or **p < 0.01, ****p < 0.0001 by one-way ANOVA **(B, D)**.

Smooth muscle cells are key proliferative contributors to femoral artery repair. Building on our initial observations, we found that the population of SMC2 was elevated during FAI (**Figure 5C**), with gene expression analyses indicating high levels of CXCL2 in this subgroup (**Figure 5E**). Inspired by Cheng’s study, which demonstrated that CXCL2 can promote hepatocyte regeneration, we investigated whether CXCL2 might similarly facilitate femoral artery repair. Immunofluorescence staining revealed that CXCL2 was highly expressed in SMCs at two weeks post-injury (FAI 2w) (**Figure 6A**). In parallel, quantitative real-time PCR (qPCR) on femoral artery samples across multiple time points (2 days, 4 days, 1 week, and 2 weeks) showed significantly elevated CXCL2 expression compared to the Sham group (**Figure 6B**). Notably, CXCL2 levels initially surged and then gradually declined, suggesting a temporally regulated role in the injury response. To further explore the mechanistic impact of CXCL2 on SMC proliferation, we used an *in vitro* model that mimics femoral artery injury. Human umbilical vein endothelial cells (HUVECs) were mechanically injured by cell scraping, and after 24 hours, the collected supernatant was mixed 1:1 with DMEM to culture SMCs (**Figure 6C**). Amodiaquine, a known CXCL2 inhibitor, was concurrently administered. CCK8 assays revealed that supernatant from injured HUVECs promoted SMC proliferation, whereas amodiaquine significantly attenuated this effect (**Figure 6D**). Notably, treatment with amodiaquine alone did not affect baseline cell proliferation, underscoring the specificity of CXCL2-mediated signaling. Collectively, these findings suggest that CXCL2 released by SMC2 fosters a self-proliferative loop, contributing to arterial repair processes following femoral artery injury.

## 4 Discussion

In this study, we re-analyzed publicly available single-cell RNA sequencing (scRNA-seq) data from a femoral artery injury (FAI) model, thereby constructing a high-resolution atlas of the cellular and molecular events underlying vascular repair. Our findings highlight the intricate roles of vascular cells—particularly smooth muscle cells (SMCs) and mesenchymal stem cells (MSCs)—and immune cells such as macrophages at different stages of arterial remodeling. Despite relying on secondary data, this integrative approach provides fresh insights into the temporal evolution of cellular heterogeneity during endovascular injury.

A central observation from our analysis is the pronounced shift in macrophage composition over time. The transition from Mac1 to Mac2 and Mac3 underscores the adaptive plasticity of the immune response, wherein early pro-inflammatory macrophages gradually give way to subpopulations that may assume pro-reparative or regulatory functions. This dynamic balance likely helps coordinate debris clearance (27), neovascularization (28), and collagen deposition (29), echoing the concept that macrophages exert stage-specific roles in tissue injury and regeneration (30). Further detailed validation—through either functional assays or *in vivo* models—would help clarify the precise cues driving these macrophage state transitions.

Our results also reveal that MSC3 expands substantially following FAI, suggesting a specialized subset with enhanced proliferative and chemokine-secreting capabilities (31). The upregulation of chemokines and cyclin-related genes in MSC3 points to a dual role in both immune cell recruitment and direct tissue remodeling, signifying its potential as a therapeutic target (32). Although these findings are derived from secondary data analysis, they align with prior studies that underscore the immunomodulatory and reparative benefits of MSCs in vascular injury. Future work involving lineage tracing and gain- or loss-of-function experiments will be critical to confirm the mechanisms by which MSC3 contributes to repair.

Smooth muscle cells play a pivotal role in arterial repair, and our subgroup analysis emphasizes the functional divergence among SMC1, SMC2, SMC3, and SMC4. Notably, SMC2 exhibits distinct inflammatory and proteolytic signatures, correlating with a unique capacity to modulate the local milieu (33). The discovery that SMC2 releases CXCL2—potentially driving its own proliferation—adds an important dimension to our understanding of how SMC subsets regulate remodeling. Inspired by Cheng et al.'s study, which demonstrated that CXCL2 can promote hepatocyte regeneration (23). And our findings suggest an analogous mechanism in vascular tissues, whereby autocrine CXCL2 signaling may accelerate SMC proliferation and neointimal formation. The effect of CXCL2 on smooth muscle cell (SMC) proliferation may be analogous to its role in hepatocyte regeneration, both of which are mediated through binding to its receptor CXCR2 and subsequent activation of the ERK signaling pathway. Inhibition of the ERK pathway may suppress the proliferation effect of CXCL2 on SMCs. Although these insights require direct experimental verification, they open up new avenues for targeted intervention to alleviate excessive proliferation and pathological restenosis of SMC.

The temporal patterns identified through pseudotime analysis further demonstrate that arterial repair proceeds in a phase-specific manner. Early time points are dominated by infiltrating immune cells that secrete pro-inflammatory mediators and orchestrate debris clearance. Over time, subpopulations such as MSC3 and SMC2 expand, suggesting a shift toward tissue rebuilding and stabilization. This stepwise progression aligns with the broader notion that successful repair depends on the tightly regulated interplay between inflammatory signaling and subsequent reparative activities.

While our re-analysis of existing scRNA-seq datasets provides valuable insights, several limitations warrant consideration. First, the data capture discrete time points rather than continuous sampling, potentially overlooking transient cell states. Second, because our conclusions are derived from secondary data, *in vivo* validation—including functional assays targeting CXCL2, MSC3, or specific macrophage subtypes—is essential to confirm causal relationships. Third, extrapolating these findings to other vascular beds or clinical settings should be approached with caution, emphasizing the need for further studies in large-animal models or patient-derived samples. Additionally, Our research is solely based on data from a single source secondary database, without conducting integrated analysis of data from other sources, which results in limitations in the representativeness of the study.

In conclusion, by interrogating a publicly available scRNA-seq dataset, our study illuminates how distinct cell subpopulations dynamically contribute to arterial repair following femoral artery injury. In particular, the macrophage plasticity, MSC3 expansion, and SMC2-driven CXCL2 autocrine signaling offer compelling mechanistic clues that could inform the design of novel therapeutics. Targeting these pathways may help optimize vascular healing and reduce complications such as restenosis or thrombosis after endovascular procedures. Moving forward, rigorous experimental validation and translational research will be key to fully harnessing these insights for improving patient care and long-term vascular health.

## 5 Conclusion

In this study, we employed single-cell RNA sequencing (scRNA-seq) to characterize cellular heterogeneity and gene expression profiles in guidewire-injured arteries, thus providing a comprehensive cell atlas of both normal and damaged arterial segments. We found that CXCL2 released by smooth muscle cells can promote its own proliferation after arterial injury. Our findings highlight key biomechanical and molecular processes involved in vascular healing, laying a foundation for future development of novel therapeutic strategies aimed at minimizing procedural damage and promoting more effective arterial repair in the context of PCI.

## Data Availability

Publicly available datasets were analyzed in this study. The datasets generated analyzed during the current study are available in the Gene Expression Omnibus (GSE182232).
